# Newcastle disease virus transmission dynamics in wild peridomestic birds in the United Arab Emirates

**DOI:** 10.1038/s41598-020-79184-3

**Published:** 2021-02-10

**Authors:** Julien Hirschinger, Lucile Marescot, Yves Hingrat, Jean Luc Guerin, Guillaume Le Loc’h, Timothée Vergne

**Affiliations:** 1 Ecole Nationale Vétérinaire de Toulouse, Institut National de Recherche Pour L’Agriculture, L’Alimentation Et L’Environnement, Unité Mixte de Recherche Interactions Hôtes Agents Pathogènes, Université de Toulouse, 31076 Toulouse, France; 2 Reneco International Wildlife Consultants LLC, PO Box 61741, Abu Dhabi, United Arab Emirates; 3 CEFE, CNRS, Université de Montpellier, Université Paul Valéry Montpellier 3, EPHE, IRD, Montpellier, France

**Keywords:** Computational models, Viral infection

## Abstract

To understand the dynamics of a pathogen in an animal population, one must assess how the infection status of individuals changes over time. With wild animals, this can be very challenging because individuals can be difficult to trap and sample, even more so since they are tested with imperfect diagnostic techniques. Multi-event capture-recapture models allow analysing longitudinal capture data of individuals whose infection status is assessed using imperfect tests. In this study, we used a two-year dataset from a longitudinal field study of peridomestic wild bird populations in the United Arab Emirates during which thousands of birds from various species were captured, sampled and tested for Newcastle disease virus exposure using a serological test. We developed a multi-event capture-recapture model to estimate important demographic and epidemiological parameters of the disease. The modelling outputs provided important insights into the understanding of Newcastle disease dynamics in peridomestics birds, which varies according to ecological and epidemiological parameters, and useful information in terms of surveillance strategies. To our knowledge, this study is the first attempt to model the dynamics of Newcastle disease in wild bird populations by combining longitudinal capture data and serological test results. Overall, it showcased that multi-event capture-recapture models represent a suitable method to analyse imperfect capture data and make reliable inferences on infectious disease dynamics in wild populations.

## Introduction

The last past decades have seen the emergence or re-emergence of many pathogenic infectious agents^1,2^. Among these latter, most are zoonoses originating from wildlife^3^. Due to their potential impact on public health, livestock health, socio-economy and wildlife conservation, understanding the dynamics of these infectious diseases has become a major research topic.

To understand the dynamics of a pathogen in a population, one must capture the changes in the infection and recovery status of individuals over time. With elusive populations such as wild animal populations, this requires setting up time- and resource-consuming longitudinal monitoring, involving capture of individuals along with the assessment of their health status by collecting biological samples and running diagnostic tests. With such protocol, an individual can remain unobserved if not captured; if captured, its health status can remain unknown if no biological sample is collected; and, if biological samples are collected, its health status can still be uncertain, due to imperfect diagnosis test leading to false positive or negative results^4^. Multi-event capture-recapture (MECR) analytical approaches represent the method of choice to deal with “imperfect” data and to assess disease dynamics in the wilderness^5,6^. Classically, these models consider discrete health states, such as “susceptible” (S), “infected” (I) or “recovered” (R), and allow adjusted estimations of various parameters, including capture probabilities, health state transition probabilities, survival rates and test performance. Recently published examples include, among others, bovine tuberculosis in badgers^7,8^, avian influenza in mallards^9^, coronavirus in bats^10^, hantavirus in rodents^11,12^, chytridiomycosis in amphibians^13–16^ or Ebola virus in gorillas^17^.

Due to its economical (direct mortality, decreased growth rate, egg drop) and ecological (impact on wild populations) importance, the Newcastle disease virus (NDV) is one of the major pathogens for captive and wild birds worldwide. Waterfowl and shorebirds are known to be natural reservoir of NDV but the host range includes numerous species, such as galliforms and columbiforms^18,19^. Non-reservoir populations are often suspected to take part in the transmission to poultry, even though their implication have been rarely and incompletely proven and documented^20^. NDV is sporadically detected in poultry in the Middle East, including the United Arab Emirates (UAE), but very little is known about its dynamics in this region^21–25^.

This study aimed at filling this gap by characterising NDV dynamics in wild birds in the UAE. We hypothesized that peridomestic species could be part of the circulation and maintenance of the virus in this specific environment and that their involvement could vary according to ecological and epidemiological parameters such as bird species, age or sampling time and site. To test this hypothesis, we used a two-year data set (January 2016–December 2017) from a longitudinal field study of peridomestic wild bird populations in the United Arab Emirates during which thousands of birds from various species were captured, sampled and tested for NDV exposure using a serological test^25^. These longitudinal data were used to fit a multi-species MECR model and estimate relevant ecological and epidemiological parameters. To our knowledge, this is the first attempt of estimating epidemiological parameters of NDV dynamics, and their source of variation, at the multi-species level in wild birds. Predictions of the model highlighted the peculiar involvement of some wild bird populations through time and space. Thus, this approach provided useful insights about the dynamics of NDV in the region and may support decision makers about wildlife management strategies.

## Material and methods

### Study area and data collection

**Figure 1:**
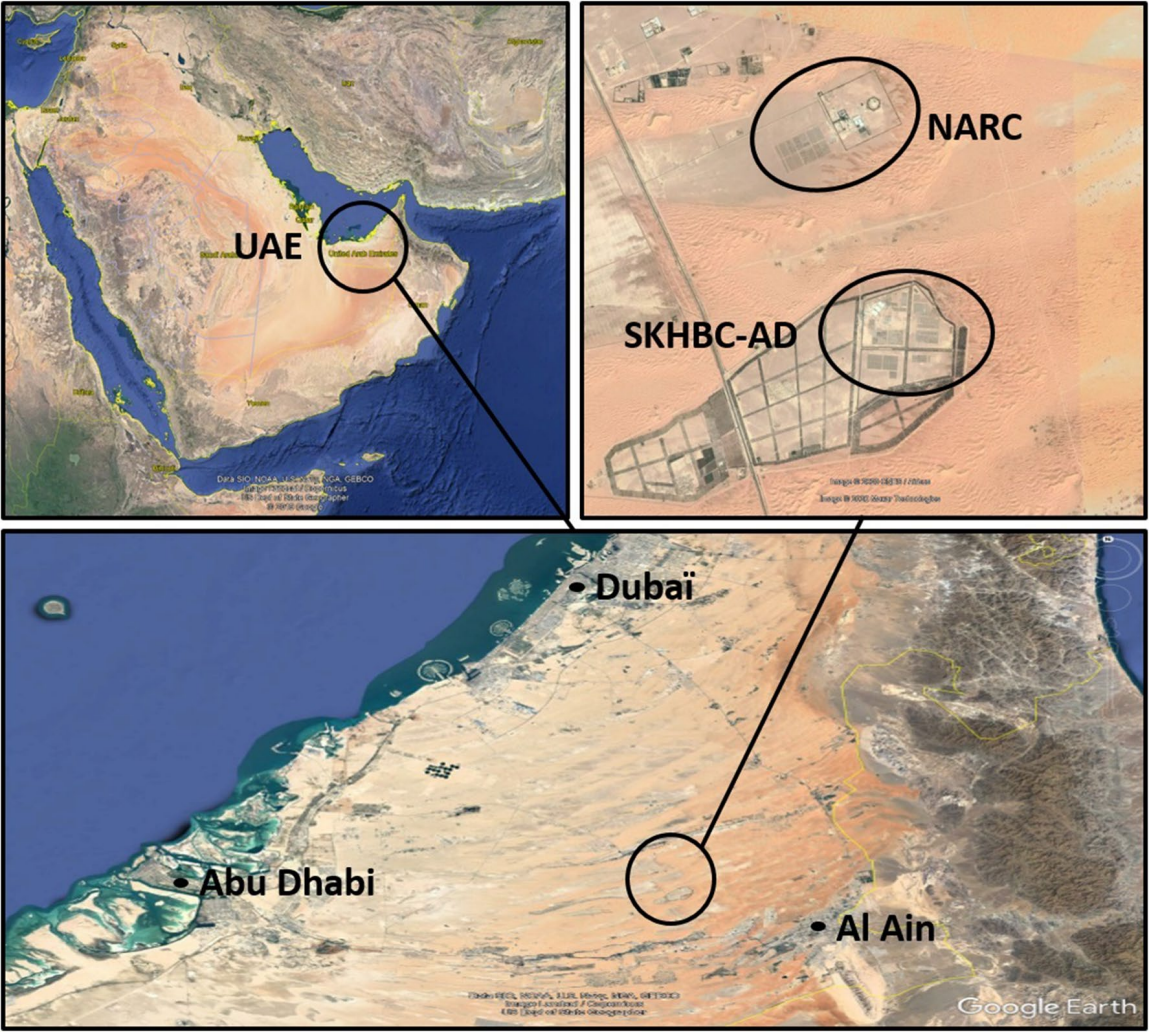
Satellite view of the United Arab Emirates. At the bottom right, the breeding sites of the National Avian Research Center (NARC) and the Sheikh Khalifa Houbara Breeding Center—Abu Dhabi (SKHBC-AD). This figure was produced on the basis of satellite views from Google Earth (Data SIO, NOAA, U.S. Navy, NGA, GEBCO, Image Landsat/Copernicus, US Dept of State Geographer @ 2019 Google, Image @ 2020 CNES/Airbus, Image @ 2020 Maxar Technologies).

This study was part of a wider project dedicated to the evaluation of sanitary risks associated with the direct and indirect contacts between captive Houbara bustards and peridomestic wild birds in the United Arab Emirates. Data were collected on two Houbara bustard conservation breeding sites, the National Avian Research Center (NARC, N24.39600 E55.43630) and the Sheikh Khalifa Houbara Breeding Center – Abu Dhabi (SKHBC-AD, N24.35400 E55.44330) both located in the Abu Dhabi emirate (Fig. 1)^25^. Breeding sites are 80 km away from the coastline, 110 kms away from Abu Dhabi, 90 from Dubaï and 30 from Al Ain. They are five kilometres away from each other. Due to a strong “oasis effect”, bird communities are thriving on these two sites, which provide resources and shelter among arid sand dunes^26^.

In each site, two trapping sessions per year have been performed in 2016 and 2017. They were organised from January to June and from September to December in order to avoid the hottest months of the year. During each trapping session, birds were captured on multiple occasions, using two main capture methods: mist nets and baited ladder letterbox traps.

According to previous demographic studies (NARC internal wild birds monitoring protocols 2008, 2011 and 2015), six species were known to be dominant in both sites and mainly involved in contacts with captive bustards: two species of passerines, the House Sparrow (*Passer domesticus*) and the White-eared Bulbul (*Pycnonotus leucotis*); three species of columbids, the Laughing Dove (*Spilopelia senegalensis*), the Collared Dove (*Streptopelia decaocto*) and the Feral Rock Pigeon (*Columba livia*) and one species of galliforms, the Grey Francolin (*Francolinus pondicerianus*). These were therefore included in the sampling protocol.

On first capture, each bird was identified to the species, sexed and aged according to Demongin^27^. Then, it was individually marked with a metal ring engraved with a unique alphanumeric code. Blood samples were collected on first capture and then, on every subsequent capture if the time interval between two consecutive samples was greater than one month. Birds were released once fully recovered from handling.

Once extracted, serum samples were stored at − 20 °C before being screened for NDV with the commercial competition ELISA kit from IDvet (ID Screen Newcastle Disease Competition) that detects antibodies targeting anti-NDV nucleoproteins.

### Capture history reconstruction

Capture histories were made of a series of four exclusive events defined at each capture occasion: “0” when the bird was not captured, “1” when it was captured but not sampled, “2” when it was captured, sampled and tested negative and, “3” when it was captured, sampled and tested positive. These events derive from the observation of the three following states: seronegative, seropositive or absent/dead. Dead encounters were also included, with individuals identified as captured but not tested (“1”) and right censored to the last encounter. Multiple captures of the same bird within a given week were aggregated into a single capture event, with the more informative event being recorded (1 < 2 < 3).

A MECR model^28^ was fitted to all capture histories, with serological status being the latent variable: the model assumed that each of the four events can correspond to any of the two serological states. The MECR model was conditional on first capture and defined by eight conditional probabilities: (i) the initial state probability, corresponding to the probability of an individual being seropositive on first capture; (ii) the survival probability, corresponding to the probability of surviving between two successive capture occasions; (iii) the two transition probabilities (conversion and reversion), corresponding to the probability of a seronegative (respectively seropositive) bird becoming seropositive (respectively seronegative) between two successive capture occasions; (iv) the capture probability, corresponding to the probability of being re-captured at a given capture occasion; (v) the probability of being tested, corresponding to the probability of a sample being taken on a captured bird and screened; and (vi) the two event probabilities (sensitivity and specificity), corresponding to the probability of a seropositive (respectively seronegative) individual testing positive (respectively negative). Moreover, the model accounted for unequal time intervals between capture occasions due to an irregular sampling calendar, with six capture occasions being more than a week away from the next one (15, 6, 3, 5, 12 and 2 weeks respectively).

### External variables

**Figure 2:**
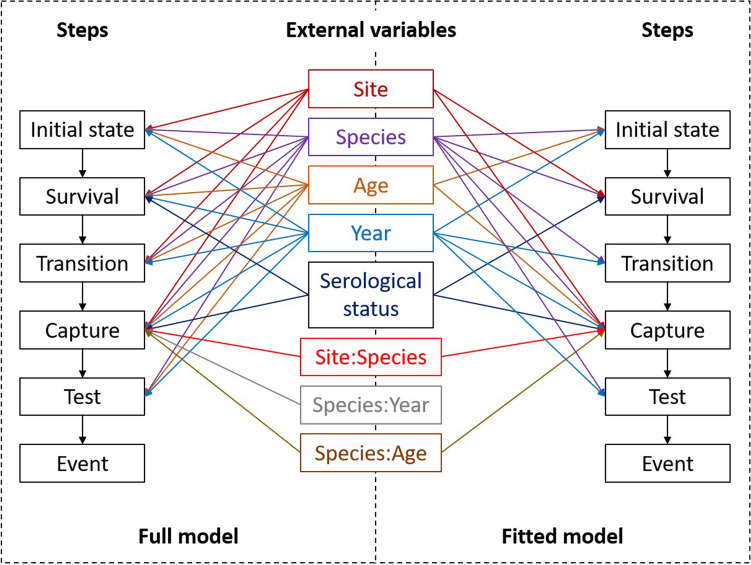
Structure of the full and best-fitted models with external variables tested. The colon “:” refers to adjacent variables that were tested in interaction.

The model was extended to account for potential variation in the different probabilities according to bird species, age (juvenile or adult), capture site (NARC or SKHBC-AD), year of sampling (2016 or 2017) and serological status. Furthermore, we also tested pair-wise interactions between these variables and the different probabilities, only if they were considered relevant a priori. The full model is depicted in Fig. 2 and includes all tested associations.

The age variable was specified in a way that led to the transition from juvenile to adult one year after the first capture if first captured as a juvenile. Bird species, age and capture site were integrated into the model as individual covariates. They were all tested on initial state, survival, transition and re-capture probabilities. As the site was not expected to affect the probability of being tested, only species and age of birds were tested on the testing probability.

An effect of the year of sampling was included in the model and tested on all probabilities since the circulation of NDV may experience temporal variations^29,30^.

As shown for other diseases, infectious status could affect the probability of survival and the probability of being captured^7,31^. Therefore, the effect of the serological status was tested on these two probabilities.

As the six dominant species were expected to be present in different proportions in the two breeding sites due to different agro-ecosystems, we tested a site-species interaction effect on the capture probability. Moreover, we also tested a species-year interaction effect on the capture probability as some species could have been more abundant on specific years. Finally, we tested a species-age interaction effect on the capture probability as some species were captured using methods leading to an age bias (i.e. pigeon chicks captured on the nests).

### Goodness of fit

There is currently no formal Goodness-of-fit (GOF) test for MECR models. Therefore, as usually done, we assessed the fit of a Cormack–Jolly–Seber model considering that model probabilities were only dependent on the species^32,33^. The software U-Care was used to this purpose^34^.

### Model selection

MECR models were fitted using the E-SURGE program version 2.1.4^35^. The simplest model was fitted to the data and significant variables were tested following a stepwise elimination procedure based on the Akaike Information Criterion adjusted for over-dispersion (QAIC), until the addition of each remaining variable increased the QAIC by more than 2 points^36^. If the difference in QAIC between models was smaller than two, the simplest one (the one with fewer parameters) was retained.

### Ethics

All birds used in this study have been captured, handled and sampled by skilled ornithologists graduated from the Centre de Recherches par le Baguage des Populations d’Oiseaux (CRBPO, Natural History Museum Paris) and trained veterinarians from the NARC according to international ethical standards^37^.

## Results

From January 2016 to December 2017, a total of 4446 different birds (1246 House sparrows, 1275 White-eared bulbuls, 978 Laughing doves, 590 Collared doves, 293 Feral rock pigeons and 64 Grey francolins) were captured and sampled during 61 capture occasions. On average, identified individuals were captured 1.2 times (range: 1–26), leading to a total of 5164 sampling occasions. During these, 3699 serum samples were collected and screened for anti-NDV antibodies. Among them, 721 tested positive and 2978 tested negative.

A detailed description of the model selection procedure is presented in Supplementary Information (Supplementary Table S1). The model with the best compromise between its likelihood and its complexity indicated that the probability of being seropositive on first capture was statistically associated with bird species, age and year of sampling. The survival probability was dependent on bird species, serological status and site of sampling. Both transition probabilities varied according to bird species and year of sampling. The probability of being re-captured was influenced by bird species, age, serological status, site and year of sampling and, the interactions between species and age, and between species and site. Finally, the probability of being tested was dependent on bird species, age and year of sampling (Fig. 2). Details of the fitted model and its outputs are presented in Supplementary Information (Supplementary Table S2) as well as all data used in this analysis (Supplementary Data S3).

GOF analysis highlighted data heterogeneity through transience and trap-dependence (X^2^ = 416.1672, df = 484, *P* = 0.99). However, the calculated variance inflation factor (Ĉ = X^2^/df = 0.86) tempered this result, suggesting a limited lack of fit. According to this analysis, four of the dominant species (the White-eared Bulbul, the Laughing Dove, the Collared Dove and the Feral Rock Pigeon) seemed to exhibit transience, meaning that some of the newly marked individuals of these species may never be seen again. In addition, all species but the Grey Francolin seemed to exhibit trap-happiness, meaning that some individuals captured on first occasion may change their behaviour and be more likely to be captured again.

**Figure 3:**
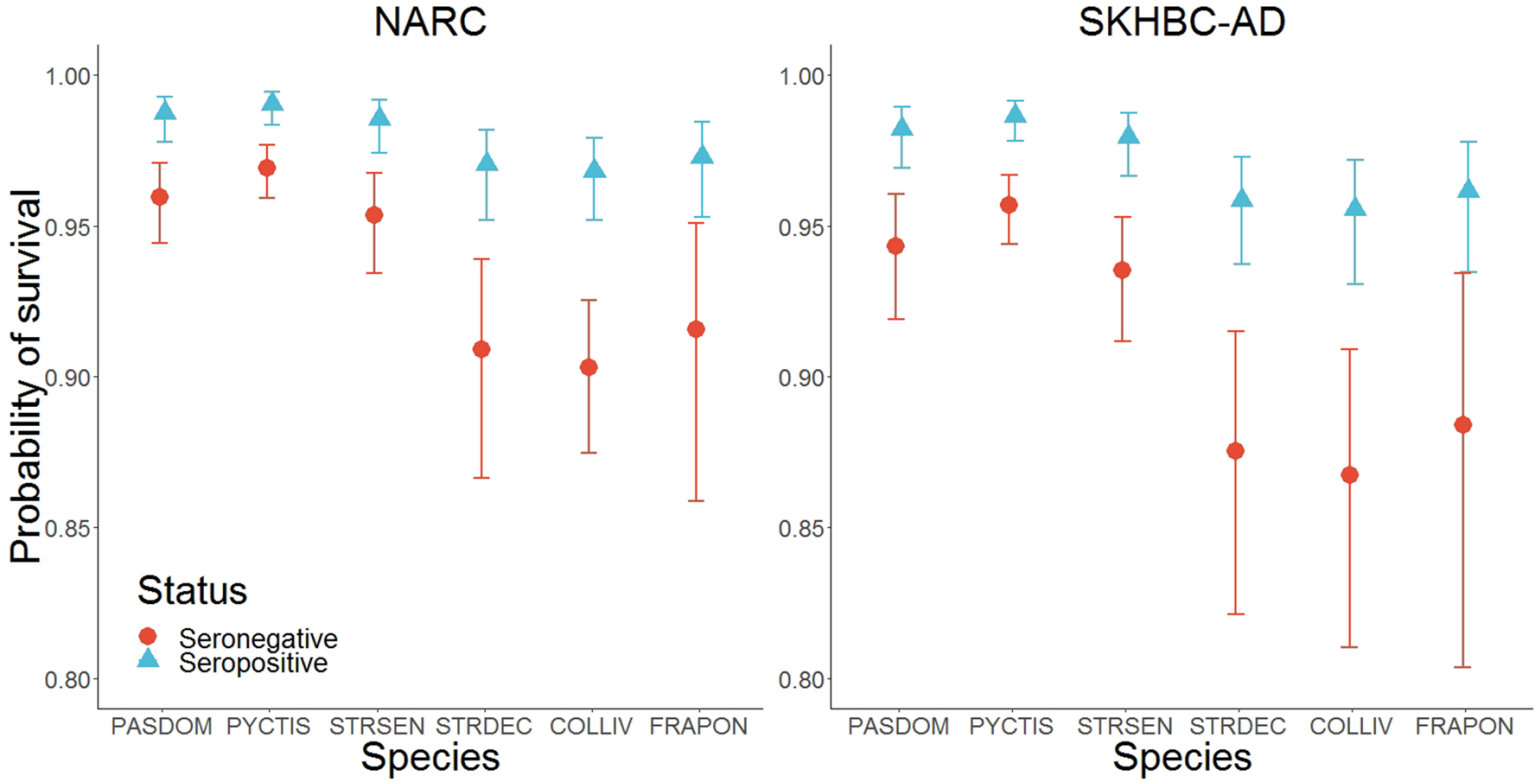
Probabilities of survival for each bird species according to the serological status of the individuals and the site of sampling. PASDOM stands for *Passer domesticus*, PYCTIS for *Pycnonotus leucotis*, STRSEN for *Spilopelia senegalensis*, STRDEC for *Streptopelia decaocto*, COLLIV for *Columba livia* and FRAPON for *Francolinus pondicerianus.* Error bars represent 95% confidence intervals.

The probability of survival between two successive capture occasions varied according to the bird species. As compared to the White-eared Bulbul, all other species presented a smaller survival probability with an odds-ratio ranging from 0.30 (95% CI: 0.20–0.44) in the Feral Rock Pigeon to 0.75 in the House Sparrow (95% CI: 0.50–1.12)*.* Site effect was also detected, highlighting a smaller survival probability in the SKHBC-AD site compared to the NARC site (OR = 0.70, 95% CI: 0.52–0.95). Finally, survival probability depended on the serological status of the individuals, where seronegative birds were less likely to survive between two successive capture occasions (OR = 0.30, 95% CI: 0.20–0.47) (Fig. 3).

**Figure 4:**
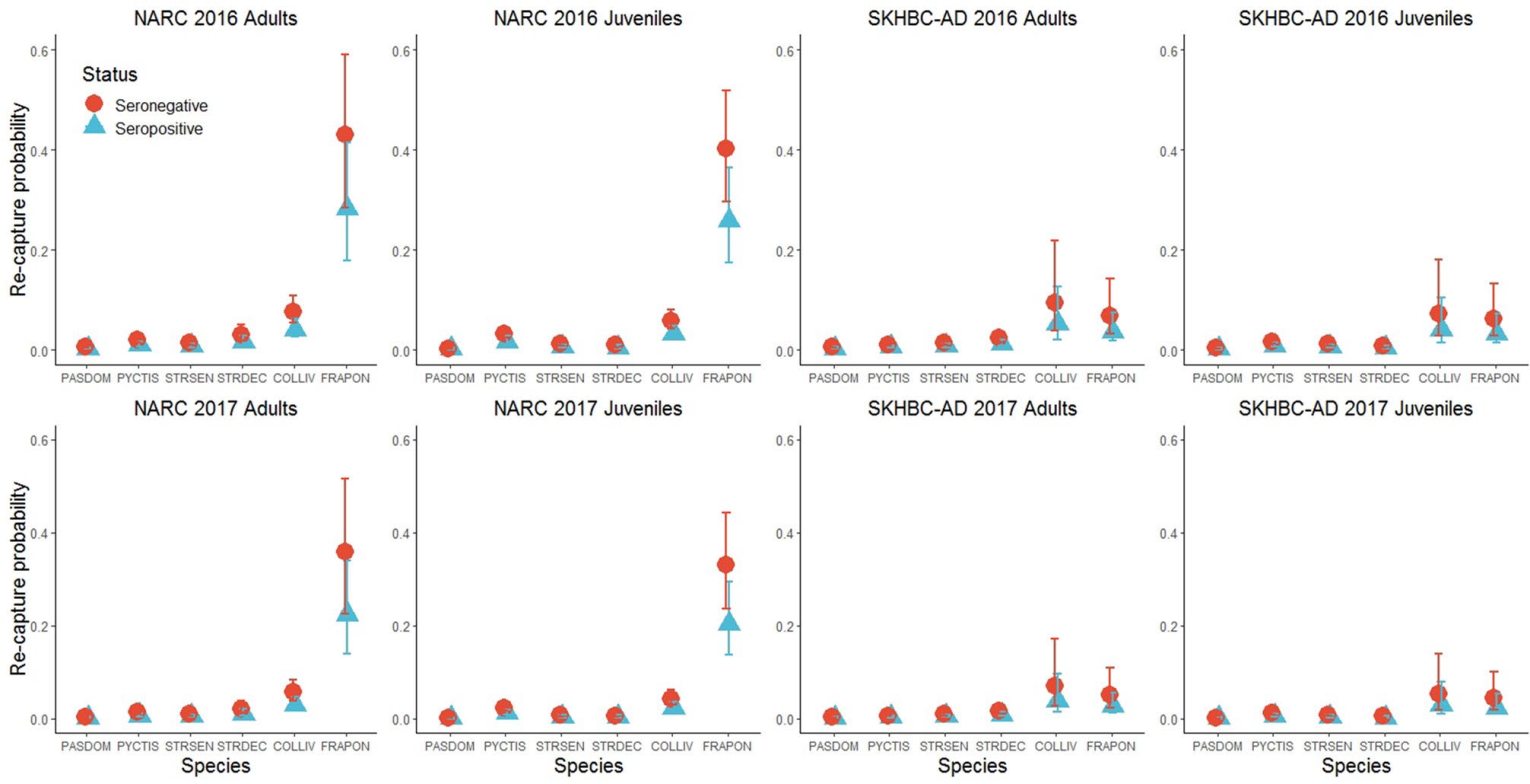
Re-capture probabilities for each bird species according to the age and serological status of the individuals and to the site and the year of sampling. PASDOM stands for *Passer domesticus*, PYCTIS for *Pycnonotus leucotis*, STRSEN for *Spilopelia senegalensis*, STRDEC for *Streptopelia decaocto*, COLLIV for *Columba livia* and FRAPON for *Francolinus pondicerianus.* Error bars represent 95% confidence intervals.

The probability of being re-captured, conditional on being alive and available for capture, depended on the serological status of the individuals and was larger in seronegative individuals (OR = 1.93, 95% CI: 1.35–2.76) than in seropositive individuals. Re-capture probability was also greatly dependent on the species, with the Grey Francolin being the most easily re-captured species. Site effect was also detected, with a larger re-capture probability in SKHBC-AD than in NARC for all species except for the White-eared Bulbul (OR = 0.52, 95% CI: 0.37–0.72) and the Grey Francolin (OR = 0.19, 95% CI: 0.08–0.44). For all species, re-capture probability was smaller in juveniles than in adults except for the White-eared Bulbul (OR = 1.70, 95% CI: 1.25 to 2.30). Age and site effects on re-capture also varied according to the species. Finally, annual variation was also detected, with a smaller re-capture probability in 2017 (OR = 0.74, 95% CI: 0.61–0.89) (Fig. 4).

**Figure 5:**
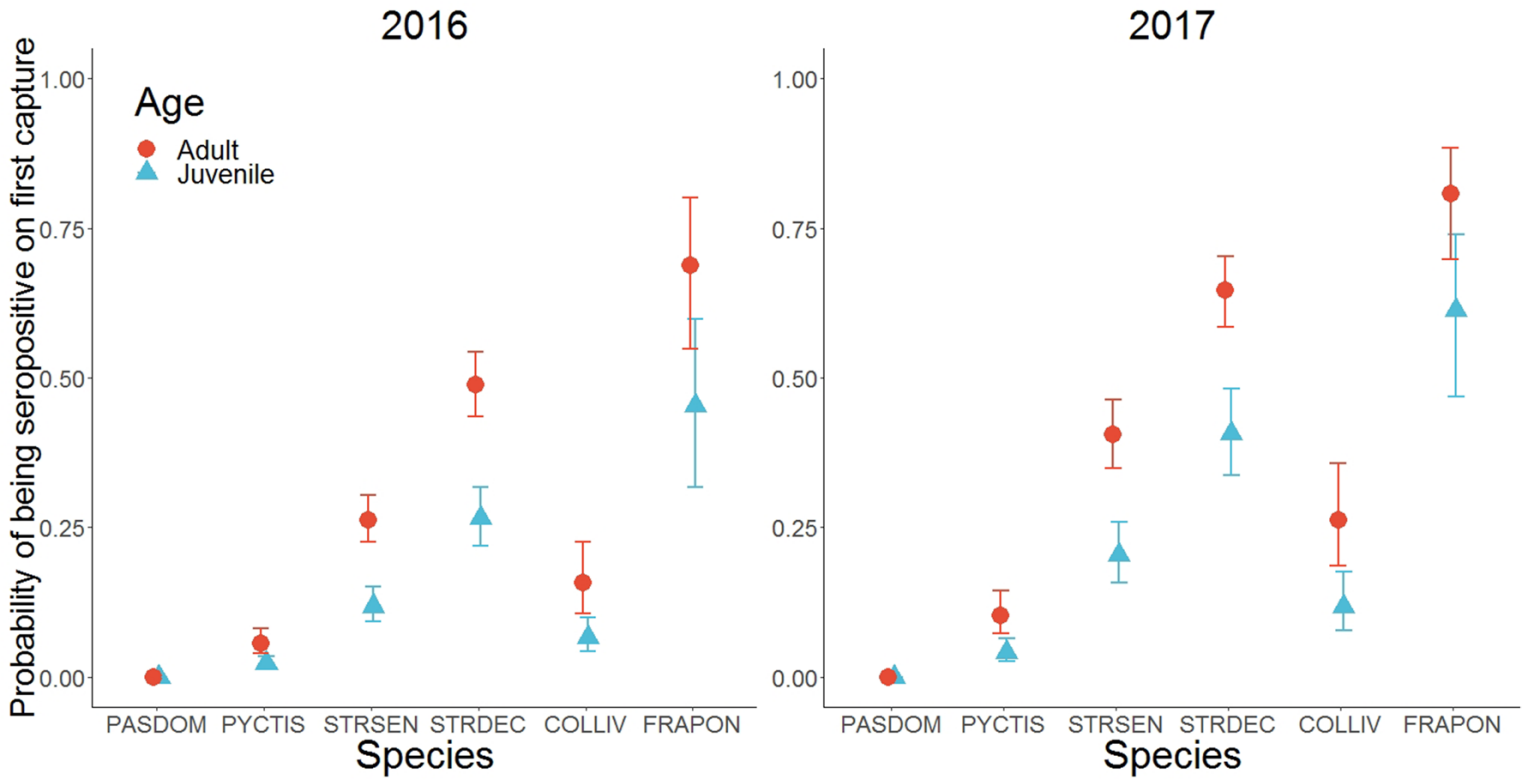
Probabilities of being seropositive on first capture for each bird species according to the age of the individuals and the year of sampling. PASDOM stands for *Passer domesticus*, PYCTIS for *Pycnonotus leucotis*, STRSEN for *Spilopelia senegalensis*, STRDEC for *Streptopelia decaocto*, COLLIV for *Columba livia* and FRAPON for *Francolinus pondicerianus.* Error bars represent 95% confidence intervals.

The probability of being seropositive on first capture was highly dependent on the bird species and was maximum for the Grey Francolin. This probability was also influenced by the age of the individuals and was smaller for juveniles (OR: 0.38, 95% CI: 0.29–0.49) than in adults. Last, being seropositive on first capture also depended on the year with a larger probability in 2017 (OR: 1.91, 95% CI: 1.51–2.41) than in 2016 (Fig. 5).

**Figure 6:**
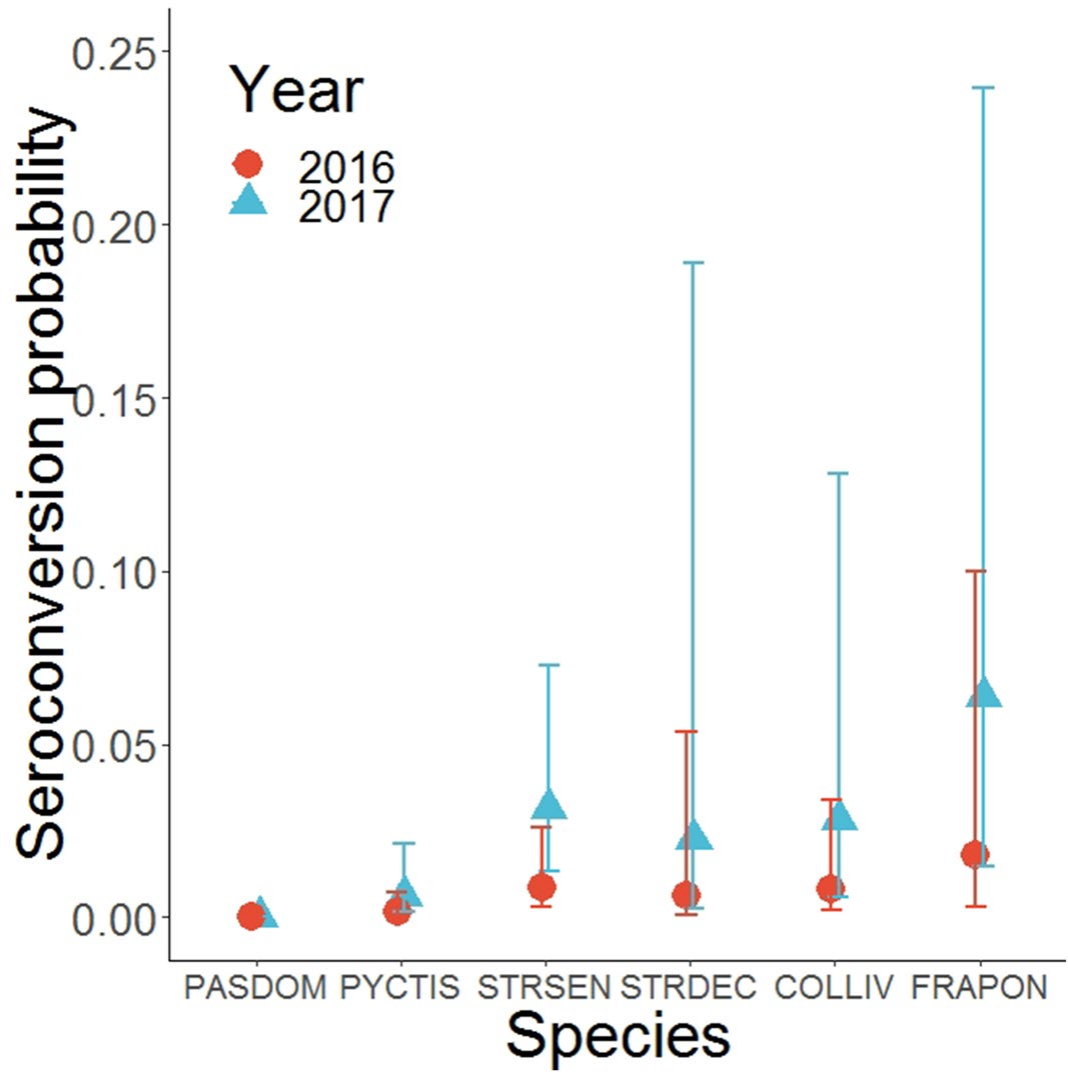
Weekly seroconversion probabilities for each bird species according to the year of sampling. PASDOM stands for *Passer domesticus*, PYCTIS for *Pycnonotus leucotis*, STRSEN for *Spilopelia senegalensis*, STRDEC for *Streptopelia decaocto*, COLLIV for *Columba livia* and FRAPON for *Francolinus pondicerianus.* Error bars represent 95% confidence intervals.

The seroconversion probability varied greatly between species. As an example, in 2017, it ranged from 9E−07% in the House Sparrow (95% CI: 2E−07 to 3.2E−06) to 6.4% in the Grey Francolin (95% CI: 1.4–23.9). Annual variation was also detected, with a larger seroconversion probability in 2017 (OR: 3.69, 95% CI: 1.06–12.84) (Fig. 6). The probability of seroreversion was estimated to be negligible for all species and years.

**Figure 7:**
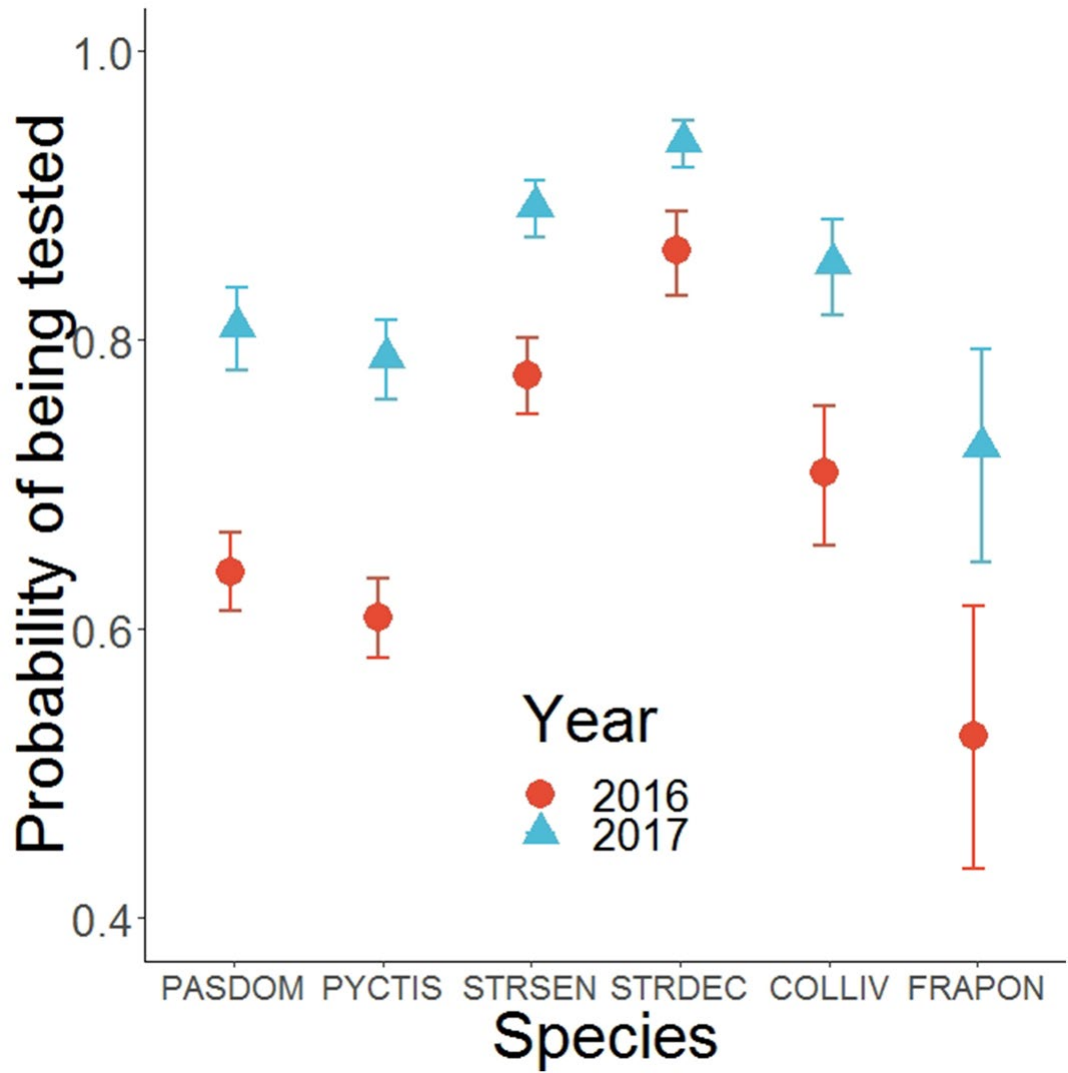
Probabilities of being tested for each bird species according to the year of sampling. PASDOM stands for *Passer domesticus*, PYCTIS for *Pycnonotus leucotis*, STRSEN for *Spilopelia senegalensis*, STRDEC for *Streptopelia decaocto*, COLLIV for *Columba livia* and FRAPON for *Francolinus pondicerianus.* Error bars represent 95% confidence intervals.

The probability of being tested varied according to the bird species, with the Collared Dove being the most frequently tested species given it has been captured. This probability was also subjected to annual variations, being larger in 2017 than in 2016 (OR: 2.39, 95% CI: 2.03–2.82) (Fig. 7).

Specificity and sensitivity of the diagnostic test was not set to previously known values but rather estimated from the MECR model. These estimations were respectively 0.97 for specificity (95% CI: 0.96–0.98) and 1.0 for sensitivity (95% CI: 1.0–1.0).

## Discussion

Individual characteristics associated with transition parameters are the main drivers of infectious diseases dynamics in wild populations. The assessment of their sources of variation is of absolute necessity when trying to predict and control the spread of infectious diseases. Although difficult to get, this information can be obtained using combined individual longitudinal pathogen screening and multi-event capture-recapture modelling. We used this method to assess the dynamics of NDV in wild peridomestics birds in United Arab Emirates. To our knowledge, this is the first attempt of estimating epidemiological parameters of NDV dynamics, and their source of variation, at the multi-species level in the wild reservoir.

Results showed that the probability of being seropositive on first capture was largely dependent on the bird species with galliforms (francolins) exhibiting the greatest probability, above columbids (doves and pigeons) and well above passerines (sparrows and bulbuls). This result is in line with literature since galliforms, especially chickens, are considered among the most likely species to be infected by NDV, whereas columbids are often infected by pigeon variants of NDV of which they are the main hosts and passerines are only rarely infected^18^. This probability was larger in adults than in juveniles, regardless of the species. This result has two plausible explanations. First, adults are more likely to have been in contact with the virus during their lifetime than juveniles. Second, in the event of NDV infection, young individuals are more likely to show clinical signs and die. Moreover, juveniles can also be unable to produce detectable antibodies as their immune system may still be immature^38,39^. Finally, the probability of being seropositive on first capture was larger in 2017 than in 2016, regardless of the species, suggesting an increased NDV circulation in 2017.

The probability of seroconversion was greatly dependent on the bird species, presenting the exact same pattern than the one highlighted in the probability of being seropositive on first capture. Seroconversion probability was also larger in 2017, corroborating the hypothesis of an increased circulation of the virus this year. According to our data, the probability of seroreversion seemed to be close to zero. Antibodies produced during a first natural infection are assumed to last for eight to twelve months, depending on the species^40,41^. Here, the data was collected over two years period, meaning that the relative absence of seroreversion is due to repeated exposure of wild birds to the virus, leading to a continuous detectable level of antibodies.

Survival probability was also influenced by the bird species, being higher in both species of passerines and in Laughing doves than in other species. This result suggest that, in the environment of the study, sparrows, bulbuls and laughing doves live longer than the other studied species what likely reflects the differences in life expectancies commonly accepted for these species: about ten years for passerines and fifteen in doves but only six and eight in pigeons and francolins, respectively^42–45^. This probability was dependent on the serological status of the individuals, with seropositive individuals exhibiting larger probability of survival than seronegative ones. This is likely to be linked to the pathological impact of the virus itself: seronegative individuals that did not encounter the virus yet are more likely to die from it whereas seropositive individuals are protected against reinfection. Furthermore, results showed that survival probability was dependent on the site of capture. Survival is considered as the outcome of biotic factors such as competition, predation or parasitism and abiotic factors such as temperature or humidity. Abiotic factors can reasonably be considered as identical in our study area and the explanation of this site-dependent variability may stand in the biotic factors including differences in resources availability and in interactions with competitors, predators and/or pathogens.

The probability of being re-captured depended on many variables, though most of them were related to methodological aspects of the study. In particular, annual effect (i.e., referring to the smaller probability of being re-captured in 2017 than in 2016) can be explained mechanically by the gradual decrease of capture effort from daily to occasional between January 2016 and December 2017. Birds’ age effect (i.e., referring to the larger probability of being re-captured in adults than in juveniles) can be explained logically as adults already accommodated to the trapping devices. Lastly, the effect of the capture site can likely be explained by the differences in the overall sizes of the sites and the bird populations housed within them. For example, NARC’s population of grey francolins is much smaller than SKHBC-AD’s one, leading to a larger re-capture probability considering the same capture effort in both sites. The probability of being re-captured was highly dependent on the bird species suggesting that some species (francolins and pigeons) are more subject to trap happiness (repeatedly captured after first capture) while others (sparrows) are more subject to trap avoidance (never captured again after first capture). Finally, the probability of being re-captured was larger in seronegative individuals than in seropositive ones what suggests behavioural changes induced by the infection, such as reduced foraging activity, as shown in previous studies^7,46^.

The probability of being tested varied according to the bird species. This is in relation to the difficulty in bleeding birds of some species, passerines being the hardest ones to take blood from. It can also be linked with the re-capture rates as the birds were not sampled if the time interval between two consecutive samples was smaller than a month, francolins and pigeons being the most frequently re-captured birds. Similarly, this probability was dependent on the year of sampling, being larger in 2017 than in 2016, linked with improvement of techniques, which is often encountered in similar studies^47^.

Test performances estimations were high, which is an unexpected result as these estimations are based on field data. However, it is in accordance with the test characteristics given by the serological kit’s manufacturer even if these are estimated on other bird species (poultry). However, in order to qualify these probabilities, we could have tested a species effect on them as serological methods are known to be sensitive to the origin of the samples screened and commercial kits are not developed for and validated on wild avian species.

The detected transience effect is most likely not linked with true seasonal migration as all target species are resident non-migratory birds, but it could be explained by juvenile dispersal. In this case, our MECR model accounts for this bias by considering an age effect on all the MECR probabilities^32,48^. The detected trap-happiness effect may be linked with the use of baited traps, for which the benefit/risk ratio is perceived as positive^49–51^. Methods to take into account trap-dependence do exist but they require to double the number of states and make the model even more complex^52^. Note that previous studies have proved that trap-dependence has only a limited effect on model results and do not particularly generate any bias on survival probability^53^.

Finally, our results provided important insights into the understanding of NDV dynamics in peridomestic birds. Particularly, they demonstrated that some of our target species, namely galliforms (francolins) and columbids (doves and pigeons), are likely to encounter the virus but, more importantly, that they may act as a reservoir considering their high seropositivity and seroconversion probabilities. These results provided useful information in terms of surveillance strategies, with the aim of detecting an increased circulation of the virus in a timely manner. Note that, in addition to the identification of the species and of the age category to target in priority (adult columbids and galliforms), the results also underlined that the capture probability was greatly dependent on the serological status, with larger capture probabilities in seronegative individuals than in seropositive ones. As emphasized for other diseases, this result is crucial as it demonstrates that observed prevalence in wild populations using a single capture occasion (cross-sectional study) can be heavily biased towards the health status of the sub-population that is more likely to be captured. This is of great importance when considering disease control, particularly control strategies based on capture-and-cull. In this case, a cull of captured individuals would result in the most likely removal of seronegative individuals, leading to the increase of the disease prevalence in the population.

## Supplementary information

Supplementary Legend.

Supplementary Table 1.

Supplementary Table 2.

Supplementary Data.
