# Supplementary material for: Newcastle disease virus transmission dynamics in wild peridomestic birds in the United Arab Emirates
Source: Sci Rep. 2021 Feb 10;11:3491. doi: 10.1038/s41598-020-79184-3 (PMC7876026; doi:10.1038/s41598-020-79184-3)
Supplement: Supplementary file 1 — Supplementary Legend. [file 41598_2020_79184_MOESM1_ESM.docx]

# **Newcastle disease virus transmission dynamics in wild peridomestic birds in the United Arab Emirates.**

# Julien Hirschinger^1,2,4^, Lucile Marescot^3^, Yves Hingrat^2^, Jean Luc Guerin^1^, Guillaume Le Loc’h^1^, Timothée Vergne^1^

1Université de Toulouse, Ecole Nationale Vétérinaire de Toulouse, Institut National de Recherche pour l’Agriculture, l’Alimentation et l’Environnement, Unité Mixte de Recherche Interactions Hôtes Agents Pathogènes, 31076 Toulouse, France

2Reneco International Wildlife Consultants LLC, PO Box 61741 Abu Dhabi, United Arab Emirates

3CEFE, CNRS, Université de Montpellier, Université Paul Valéry Montpellier 3, EPHE, IRD, Montpellier, France

4Corresponding author (email: [julien.hirschinger@envt.fr](mailto:julien.hirschinger@envt.fr))

**Supplementary Information**

Supplementary Table S1. Model selection procedure.

Supplementary Table S2. Fitted model details and outputs.

Supplementary Data S3. E-SURGE data.
